# Status of Day Care Laparoscopic Appendectomy in Developing Countries

**DOI:** 10.1155/2014/502786

**Published:** 2014-07-10

**Authors:** Akhlak Hussain, Sarabjit Singh, Kuldip Singh Ahi, Mohinder Singh

**Affiliations:** ^1^Akhlak Hussain: Safdarjang Hospital, New Delhi 110029, India; ^2^Sarabjit Singh, Kuldip Singh Ahi and Mohinder Singh: Rajindra Hospital, Patiala, Punjab 147001, India

## Abstract

The practice of laparoscopic appendectomy as an ambulatory surgery is uncommon even in apex institutes, more so in developing countries, despite proven feasibility. To promote this practice in the developing countries like ours, we attempted to find the safety and cost effectiveness in such institutions which have limited resources. Thirty cases of symptomatic appendicitis were tried for same day discharge after laparoscopic appendectomies. The results were encouraging with 87% patients discharged on the same day and 13% on the next day in the early morning. Among the next day discharged cases, only 03% stayed for medical reasons (nausea, vomiting, and pain) while 10% stayed as their attendants declined to leave (social reasons), even though they were medically eligible for discharge from the hospital. There were no significant postoperative complications except tolerable pain in all patients and mild to moderate nausea/vomiting in 80%. There was no readmission. The mean length of hospital stay was 11.20 hrs. At the time of discharge all patients were highly satisfied. We concluded that routine same day discharge is safe and feasible after appendectomies in developing countries, with social decline as the main hurdle which can be improved by proper communication.

## 1. Introduction

Being one of the common gastrointestinal surgical emergencies, appendicitis consumes a significant portion of our hospital resources and affects the routine life of the entire family of the patient. If we can reduce the hospital stay and send the patient home as early as possible without sacrificing safety, we could minimize the use of hospital resources and the disruption of the household. The ability to provide high quality and cost effective care has made outpatient surgery one of the fastest growing areas in our health care delivery system and the ability to perform more extensive operations on an outpatient basis has focused increasing interest on outpatient (ambulatory) anaesthesia. Simultaneously, laparoscopy has fulfilled the requirements of day care surgery. To the best of our knowledge, no formal trials of the efficacy and safety of day care laparoscopic appendectomy have been performed in tertiary government institutions in our country where proper staff and setup are not available. We proposed to standardize same day discharge after laparoscopic appendectomy, making it the usual and customary pathway. We studied prospectively the outcomes of and patient satisfaction with day care laparoscopic appendectomy.

## 2. Methods

After institutional review board approval, patients with uncomplicated appendicitis were considered for day care laparoscopic appendectomy (Table 1). All surgeries were done the day after the admission in morning either as first or second case. Informed consent after full explanation of day care surgery process was taken.

The protocol included premedication with Ramosetron (0.3 mg) i.v., Midazolam (2 mg), and Phenergan (25 mg) i.m., 30 minutes before surgery. Induction was done by Glycopyrrolate (0.2 mg) i.v., Fentanyl (3 micro gm/kg) i.v., and Propofol (1–1.5 mg/kg) i.v.. Relaxation was rendered by Atracurium (0.3–0.5 mg/kg) i.v.. Maintenance was done with O_2_, N_2_O, and Isoflurane. Atracurium and Fentanyl were given when required. Regular monitoring of hemodynamic parameters including pulse rate, blood pressure, oxygen saturation, and electrocardiogram was done. Surgical approach included three ports (one 10 mms and two 5 mms). If required blunt dissection was used to identify appendix. After ligation of the base, appendix was divided and delivered through umbilical port. Stump was cleaned, peritoneum was deflated, trocars were removed, and incision was closed aseptically. Oral liquids were allowed after 6 hours of surgery. Oral or rectal NSAIDS and intravenous or oral ondansetron were used as per requirements. Postoperatively patient was monitored for vitals, postoperative complications, morbidity, total hospital stay, and complications in follow-up.

*Criteria for Discharge*

- Stable vital signs for >30 min.
- No new signs or symptoms after the operation.
- No active bleeding or oozing.
- Minimal nausea and persistent emesis for <30 min.
- Orientation to person, time, and place.
- Pain controllable with oral analgesics.
- Passed urine.
- No surgical complication.
- Minimal dizziness after sitting for <10 min.
- A responsible escort.

All patients were provided a set of instructions regarding diet and report in case of fever, nausea, vomiting, excessive pain, constipation, diarrhea, and wound site discharge or redness. Overnight stay was considered in cases of improper recovery, any complications (e.g., vomiting and uncontrolled pain), and social issues (transportation problem or family's will to stay). Patient's demographics, duration of surgery, length of stay after surgery, postdischarge visit in emergency, readmission, and complications were collated.

## 3. Results

All patients who underwent laparoscopic appendectomy were found eligible for same day discharge, except for one patient. Of the total, 87% were discharged on the same day with the average postoperative length of 9.62 hrs (range: 7 to 12). While only 13% were discharged on next day in the morning with the average postoperative length of stay of 22 hrs (range: 20 to 23). Among the next day discharged cases, 3% stayed for medical reasons (nausea, vomiting, and pain) while remaining 10% had to stay as their attendants declined to leave (social reasons, i.e., poor understanding and fear), even though they were medically eligible for discharge from the hospital. Overall the mean length of hospital stay was 11.20 hrs (range: 7 to 23). Average length of surgery was 55.50 min (range: 35 to 80). There were no significant effect of duration of surgery regarding postoperative complications and duration of ambulation after surgery. All four patients who stayed overnight had duration of surgery ≤51 min (which is significant). This shows that although prolonged surgery can delay discharge, there are other significant factors which affect the day care surgery.

73% patients were ambulated within 6 hours and given oral medications thereafter while 27% were ambulated after 6 hrs because of vomiting, pain, and less motivation. There were no significant postoperative complications except pain in all patients (VAS score ranging from 1 to 4 only) and mild to moderate nausea/vomiting in 80%. At the time of discharge all patients (100%) were highly satisfied. Follow-up was scheduled on 2nd, 5th, and 10th day postoperatively. There was no urgent postoperative visit other than the scheduled follow-up. During follow-up, fifteen patients complained of pain (VAS-1 and 2) for 2 days and a single patient complained of pain (VAS-2) for 5 days. There was no readmission. Fifteen patients returned to their full routine activities within 3 days and remaining started doing their full activities within 7 days (Table 2).

## 4. Discussion

We set out to determine the feasibility of offering laparoscopic appendectomy as a day care procedure. Our early results are encouraging and indicate that such an offer is practical. Appropriate patient selection lowers the failure rate. Usually, patients with ASA grades I, II, and well controlled ASA III are selected for day care procedures. We also preferred the same and this resulted in successful adaptation of day care laparoscopic appendectomy in 100% of patients. In our study, unplanned readmission or follow-up rate was zero. This was possible due to proper patient selection. In the study of Schreiber, 78 cases of clinically acute or subacute appendicitis were tried with outpatient laparoscopic appendectomy. Patients with severe disease presenting with sepsis or peritonitis were excluded. Five postoperative complications (four cases of peritonitis and one stump insufficiency) were found and treated by laparotomy [1]. In the study of Brosseuk and Bathe, two (4%) of the fifty-two patients who underwent laparoscopic appendectomy had significant complications, one of them required reoperation for intra-abdominal abscess. Thirty-nine (75%) of the laparoscopic appendectomies were done as day care procedures [2]. Alvarez and Voitk found that there were no readmissions for wound infections or postoperative abdominal abscesses. They concluded that over one-half of patients with complicated appendicitis can be managed as outpatients without jeopardy to outcome [4]. In the study by Bensard et al., overall 66% of the children with acute appendicitis (27/41) and 27% with suppurative appendicitis (3/11) were discharged within 24 hours of admission [6]. They found that no patients in these groups had suffered a complication or were readmitted following discharge (Table 3).

For the success of day care surgery, familiarity with the procedure is essential. Our team has perfected the technique and had performed over 100 such procedures. Currently, our mean operating time is 51 min (range 35–80 min). In the study of Alkhoury et al., operative duration was averaging 23 min (range, 6–61 min) in the same day discharge group versus 22 min (range, 10–77 min) in the overnight admission group (*P* > 0.05) [8]. In the present study, overnight stay occurred in cases with length of operation lesser than our average duration. Thus, it can be concluded that in surgeries of duration less than one and half hours, the duration of surgery does not significantly affect the timing of discharge.

Overnight stay is usually a joint decision made by the surgeon, the patient, and his attendants. As patient has to participate in self-care after discharge, their comfort, preference, and safety need to be considered in the assessment for discharge. In our study, four patients were admitted overnight. Out of them, only one stayed due to medical indications and the other three stayed when their attendants declined to leave (social reasons), even though they were medically eligible for discharge from the hospital. The higher rate of overnight admission due to social reasons explained the fear and lack of proper knowledge among the people of lower socioeconomic status which forms the main bulk of our type of government setup. In the study of Alkhoury et al., 45 (out of 207) children were admitted overnight because the hour was too late for discharge in 35 (77.8%), medical indications dictated admission in 5 (11.1%), and social reasons required admission in 5 (11.1%). The complication rates were similar in the same day discharge group (8.0%) and in the admitted group (6.6%), as were the rates of urgent postoperative visits (7.4% versus 4.4%%) and the readmission rates (2.5% versus 2.2%) (*P* > 0.05 for all) [8].

Many series have documented a decreased incidence of postoperative complications and a decreased incidence of wound infection after LA [9–11]. In our series, no patient developed any significant complication. Certainly, the laparoscopic approach facilitates the complete inspection of the abdominal cavity and identification of all septic foci or any significant pathology. Thus, laparoscopic approach increases the precision of diagnosis, avoiding additional complications.

It has been suggested that, with increasing operative experience, the operative time required for LA will decrease significantly [10]. The use of nondisposable laparoscopic equipment significantly decreases the cost of LA [9–12]. Lastly, it has been suggested that even if the patients are not discharged from the hospital soon enough after LA to make a significant difference between the cost of LA versus OA, LA has a much shorter recovery time and returns patients to a productive lifestyle sooner, thus justifying LA [9]. Overall, the DCLA is more cost effective as compared to traditional inpatient cases. The main cost savings were in bed costs (general ward, semiprivate, or private) and institutional data; however, operative procedure cost was similar in both cases. In the present study, which was conducted in government hospital where bed charge, nursing charges, and overall hospital charges are minimal, significant cost savings are not noticed. But, early return of productivity saved wages of 2-3 days. It was inferred that cost saving is more effective in private setup where hospital charges are higher.

The findings of the present study regarding the effectiveness of laparoscopic appendectomy as day care procedure are consistent with previous researches. Our results demonstrated that day care laparoscopic appendectomy is safe with high success rate in carefully selected patients with uncomplicated appendicitis and has the advantage of cost effectiveness.

## 5. Conclusion

The present study was done to demonstrate the safety of laparoscopic appendectomy in a day care setting in a government hospital attached to a medical college in India. The following conclusions were drawn.

1. Day care laparoscopic appendectomy is eminently feasible in government hospital settings in India.
2. Anesthesia requirements include premedication for anxiolysis and sedation and to prevent postoperative nausea and vomiting, general anesthesia with rapidly acting drugs having high clearance rate, minimal side effects, and rapid recovery.
3. Most of the patients can be discharged by the evening of the same day, thus avoiding overnight hospital stay (average duration of stay is 9.62 hrs). Only one of our thirty cases (03%) needed to be kept in the hospital overnight for medical reasons (nausea, vomiting, and pain).
4. A curious feature of Indian scenario is that some patients insist on staying in the hospital overnight for social reasons as their attendants feel uncomfortable on leaving hospital so early. Thus, three (10%) of our patients had to be kept in the hospital overnight, although they could have been discharged safely.
5. Proper selection prevents unplanned readmission and follow-up, thereby increasing success rate.
6. For surgeries of duration less than one and half hours, the duration of surgery does not significantly affect the timing of discharge. There are many other factors which affect the day care surgeries. Among them, social fear and reluctance is one of the important factor.
7. Finally, we advised further studies to develop stronger recommendations, as our study sample was small.

## Figures and Tables

**Table 1:** Patient selection criteria.

Inclusion	Exclusion
Uncomplicated symptomatic appendicitis, that is, without abscess, perforation, sepsis, and phlegmon formation. Medically fit and stable patients {ASA I, II, III (well controlled)}. Well motivated and psychologically/mentally stable.	Multiple comorbid diseases, coagulation disorders, and adverse anaesthetic history. Suspected/proven malignancy. ASA III (uncontrolled) or IV. Unavailability of competent adult to accompany the patient. Age <14 and >60years. Body mass index >35. Long distance from home (> 30min travel)

**Table 2:** Comparison of duration of discharge.

Parameters	Same day discharge	Overnight stay	Inference
Number of patients	26	4	—

Number of patients with postoperative nausea, vomiting, or pain with VAS ≥2.	22	4	—

Number of patients with VAS ≥2 during discharge	5	2	—

Average time of postoperative ambulation after surgery (hrs)	5.73 (range 4–7)	6.75 (range 6–7)	*P* = 0.0246^*^

Average length of hospital stay (hrs)	9.62 (range 7–12)	22.00 (range 20–23)	*P* = 0.0001^*^

Number of patients with Nausea, vomiting, or pain with VAS ≥2 in follow-up	2	2	—

Readmissions	0	0	—

Emergency follow-up	0	0	—

Number of satisfied patients	26	4	—

^*^Statistical significant two-tailed *P* value (unpaired *t* test results).

**Table 3:** Day care appendectomies.

Author	Study design	Types and number of patients	Appendectomies	D/C < 24 hrs	RR
Schreiber [1]	Retrospective	Acute and subacute appendicitis *n* = 78	Laparoscopic	Outpatient, 100%	5

Brosseuk and Bathe [2]	Retrospective	Acute appendicitis *n* = 52	Laparoscopic	75%	—

Velhote et al. [3]	Prospective	Appendicitis *n* = 144	Open	86%	2

Alvarez and Voitk [4]	Case series	Perforated appendicitis *n* = 38	Laparoscopic	57%	Nil

Pfeil et al. [5]	Case series	Appendicitis *n* = 56	Open	100%	—

Bensard et al. [6]	Case series	Appendicitis *n* = 72	Laparoscopic	66% (acute appendicitis) 27% (suppurative appendicitis)	Nil in both cases

Gilliam et al. [7]	Case series	Acute appendicitis	Laparoscopic	72%	—

Alkhoury et al. [8]	Case series	Appendicitis *n* = 207	Laparoscopic	78.3% (same day)	2.2–2.5%

Present study	Case series	Acute appendicitis	Laparoscopic	100% 87% (same day)	Nil

(D/C: discharge; RR: readmission rate).
